# Inflammatory cytokine profile in patients with sickle cell anaemia and leg ulcers

**DOI:** 10.1111/bjh.70236

**Published:** 2025-11-03

**Authors:** M. V. Diniz, A. P. Silva, G. S. Arcanjo, T. H. C. Batista, J. V. S. Rodrigues, M. J. B. M. Rêgo, H. I. Leão, R. C. Silva, F. O. Souto, C. M. L. Melo, B. R. S. Barros, A. C. M. Anjos, A. S. Araújo, I. F. Domingos, S. T. O. Saad, F. F. Costa, A. R. Lucena‐Araujo, M. A. C. Bezerra

**Affiliations:** ^1^ Genetics Postgraduate Program Federal University of Pernambuco Recife Brazil; ^2^ Center of Research in Therapeutic Innovation (NUPIT‐UFPE) Recife Brazil; ^3^ Aggeu Magalhães Institute/Oswaldo Cruz Foundation Recife Brazil; ^4^ Laboratory of Immunological and Antitumor Analysis (LAIA) Keiso Asami Institute of Immunopathology (iLIKA‐UFPE) Recife Brazil; ^5^ Department of Internal Medicine Hematology and Hemotherapy Foundation of Pernambuco (HEMOPE) Recife Brazil; ^6^ Cardiology Emergency Unity of Pernambuco (PROCAPE) University of Pernambuco Recife Brazil; ^7^ Hematology and Hemotherapy Center State University of Campinas São Paulo Brazil

**Keywords:** hydroxycarbamide, interleukins, NLRP3, sickle cell disease, skin wounds

To the Editor,

Sickle cell anaemia (SCA) is characterised by variable clinical complications, including leg ulcers (LUs). LUs typically develop in areas with reduced subcutaneous tissue, thin skin and impaired blood flow. These lesions are recurrent, heal slowly and currently lack definitive treatments.^1^, ^2^, ^3^ Their pathological progression is closely associated with chronic intravascular haemolysis and can be influenced by environmental factors, social conditions and genetic predispositions.^4^, ^5^ Recent studies indicate that SCA patients with LUs exhibit elevated levels of inflammatory cytokines, underscoring the role of inflammation in these lesions.^6^, ^7^ In this context, the analysis of serum cytokine levels may provide valuable insights into LUs' pathophysiology as well as potential therapeutic targets.

In the present study, we evaluated the serum levels of interleukin (IL)‐1β, IL‐6, IL‐8, IL‐10, IL‐12p70, IL‐18 and tumour necrosis factor‐alpha (TNF‐α) in 76 adult SCA patients (homozygous haemoglobin SS genotype), all over 18 years of age, who attended the Haematology and Haemotherapy Centre in Northeast Brazil. Detailed methodological procedures are described in Data S1. According to the clinical history of LU at the time of blood sample collection, patients were stratified into active LU (ALU), healed LU (HLU) and the haemoglobin SS control (Control‐HbSS) groups. Detailed clinical and laboratory data are presented in Table S1. Additionally, we included a group of 20 individuals with normal haemoglobin profile (Control‐HbAA) for comparison.

We first compared the serum cytokine levels among the LU group, Control‐HbSS and Control‐HbAA. The LU group consisted of SCA patients with active and healed LU, who were pooled together based on observed similarities in their clinical and laboratory features. LU patients showed higher TNF‐α and IL‐12p70 compared to both Control‐HbSS and Control‐HbAA groups. Compared to Control‐HbAA, LU patients additionally have elevated IL‐6, IL‐8, IL‐10 and IL‐18 (Figure S1). To determine the influence of hydroxyurea (HU; hydroxycarbamide) therapy on cytokine profiles, LU patients were divided into those receiving HU and those not receiving HU and compared to HU‐naive Control‐HbSS. TNF‐α remained elevated in LU patients regardless of HU treatment. Moreover, IL‐12p70 was higher in LU HU‐treated patients compared to HU‐naive HbSS controls, and IL‐10 was higher in HU‐treated patients compared to both untreated patients and controls (Figure S2).

Subgroup analysis was conducted to assess cytokine levels among patients with ALU, HLU and Control‐HbSS. TNF‐α was significantly elevated in ALU (*p* = 0.0002) and HLU (*p* = 0.043) compared to Control‐HbSS. IL‐8 was significantly higher in HLU than in Control‐HbSS (*p* = 0.037). IL‐10 levels were elevated in ALU compared to Control‐HbSS (*p* = 0.036), and IL‐12p70 was higher in HLU compared to Control‐HbSS (*p* = 0.0014). IL‐1β, IL‐6 and IL‐18 levels did not differ significantly among the three groups (Figure 1A–G).

**FIGURE 1:**
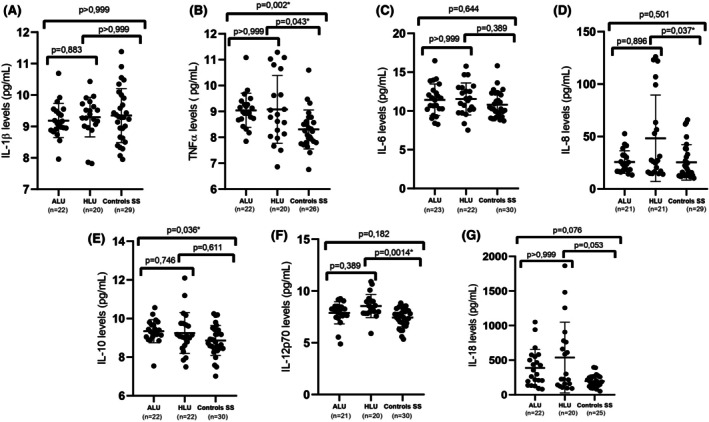
Cytokine levels in patients with active leg ulcers (ALU), healed leg ulcers (HLU) and controls with sickle cell anaemia (ControlsHbSS). Serum concentrations of IL‐1β (A), TNF‐α (B), IL‐6 (C), IL‐8 (D), IL‐10 (E), IL‐12p70 (F) and IL‐18 (G) were measured and compared among patients with ALU, HLU and control HbSS. Data are presented as individual values with mean and standard deviation. Statistical comparisons were performed using the Kruskal–Wallis test followed by Dunn's multiple comparisons post‐test. Exact *p*‐values are indicated; *p < 0.05 was considered statistically significant. IL, interleukin; TNF‐α, tumour necrosis factor‐alpha.

To further explore cytokine level patterns among groups, partial least squares discriminant analysis (PLS‐DA) was performed. A partial separation between the groups according to the cytokine profile was observed, particularly for the HLU group relative to the Control‐HbSS and ALU groups, suggesting that cytokine levels vary subtly according to ulcer status (Figure 2A). The variable importance in projection (VIP) scores ranked cytokines according to their contribution to group discrimination. TNF‐α and IL‐8 exhibited the highest VIP scores, indicating their role in distinguishing the clinical groups. IL‐1β and IL‐10 also contributed substantially, while IL‐18, IL‐12p70 and IL‐6 displayed lower importance (Figure 2B).

**FIGURE 2:**
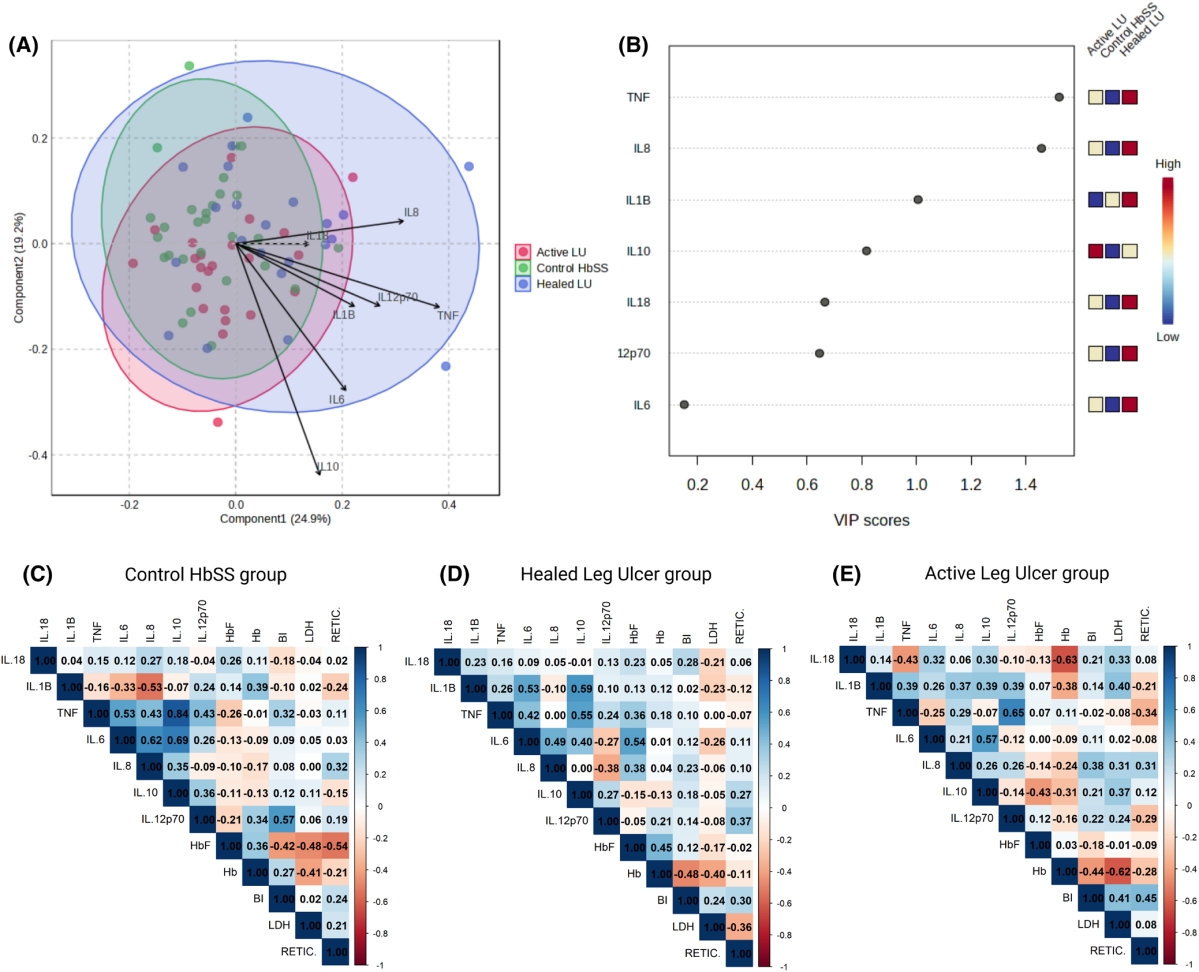
Partial least squares discriminant analysis (PLS‐DA), variable importance in projection (VIP) scores and Spearman correlation analysis of cytokine profiles. (A) The PLS‐DA score plot shows the separation of individuals with active leg ulcers (red), healed leg ulcers (blue) and Control HbSS (green) based on their cytokine profiles. Ellipses represent 95% confidence intervals for each group. (B) VIP scores derived from the PLS‐DA model ranked the cytokines according to their importance in differentiating the groups. (C–E) Spearman's correlation coefficients for cytokines (IL‐18, IL‐1β, TNF‐α, IL‐6, IL‐8, IL‐10, IL‐12p70) and laboratory parameters across study groups. The laboratory parameters include fetal haemoglobin (HbF), haemoglobin concentration (Hb), indirect bilirubin (BI), lactate dehydrogenase (LDH) and reticulocyte count (RETIC) in (C) Control HbSS, (D) healed leg ulcer and (E) active leg ulcer groups. IL, interleukin; TNF, tumour necrosis factor.

A correlation analysis was performed to analyse the association between the cytokines and haemolytic markers. In the HbSS control group, IL‐1β was negatively correlated with IL‐8 (*p* = 0.011). TNF‐α exhibited positive correlations with IL‐6 (*p* = 0.011), IL‐8 (*p* = 0.045), IL‐10 (*p* < 0.0001) and IL‐12p70 (*p* = 0.045). IL‐6 was positively correlated with IL‐8 (*p* = 0.002) and IL‐10 (*p* < 0.001). IL‐12p70 showed a positive correlation with indirect bilirubin (IB) (*p* = 0.005) while fetal haemoglobin (HbF) was negatively correlated with IB (*p* = 0.050), lactate dehydrogenase (LDH) (*p* = 0.024) and reticulocyte count (*p* = 0.010) (Figure 2C). In individuals with HLU, IL‐1β demonstrated significant positive correlations with IL‐6 (*p* = 0.017) and IL‐10 (*p* = 0.006). TNF‐α was positively correlated with IL‐10 (*p* = 0.012), and IL‐6 showed a significant positive correlation with HbF (*p* = 0.015). Haemoglobin levels were inversely correlated with IB (*p* = 0.032) (Figure 2D). Finally, in the ALU group, TNF‐α showed a strong positive correlation with IL‐12p70 (*p* = 0.002), and IL‐6 was also positively correlated with IL‐10 (*p* = 0.009). Among haematological markers, IL‐18 was negatively correlated with Hb (*p* = 0.003) and Hb levels were inversely correlated with LDH (*p* = 0.004). Additionally, reticulocyte count showed a positive correlation with IB (*p* = 0.047) (Figure 2E).

Our findings revealed a distinct pro‐inflammatory pattern in the LU group, suggesting an intensified inflammatory response associated with these lesions. The analysis of inflammatory cytokines revealed significant elevations in TNF‐α and IL‐12p70 levels in LU patients compared to the control HbSS group. Notably, TNF‐α and IL‐8 emerged as key discriminators of LU status, as reflected by their high VIP scores in the PLS‐DA analysis. These cytokines are well‐known mediators of inflammation, tissue injury, wound healing, chronic ulceration and their elevated levels suggest that they may play critical roles in the pathophysiology of LUs in SCA.^8^ TNF‐α is a central mediator of inflammation, promoting leucocyte recruitment, endothelial activation and tissue damage, all of which are critical processes in the development and persistence of chronic ulcers.^5^, ^9^ Elevated TNF‐α is described to impair angiogenesis and inhibit fibroblast and keratinocyte proliferation and migration in diabetic wounds.^10^

Elevated IL‐12p70 may contribute to sustained inflammatory signalling in these patients, potentially exacerbating local tissue injury and impairing wound resolution.^5^ IL‐8, as a potent neutrophil chemoattractant and pro‐angiogenic factor, is known to contribute to revascularisation and matrix remodelling in healing tissues.^5^, ^11^ High levels of IL‐8 in SCA patients with LUs were previously reported.^6^, ^7^ Interestingly, TNF‐α, IL‐12p70 and IL‐8 levels were notably increased in the HLU group compared to the control HbSS group. Its persistent elevation post‐healing suggests that these reparative mechanisms may continue beyond visible wound closure, or that underlying subclinical inflammation persists. The persistence of inflammation in HLU patients suggests a chronic inflammatory state that may predispose them to recurrence, highlighting the need for long‐term management strategies.

HU, a key therapy in the management of SCA, did not fully alleviate the inflammatory state associated with LUs in this study. Patients with LUs undergoing HU therapy continued to exhibit elevated levels of IL‐12p70 and TNF‐α compared to the control HbSS group, indicating persistent inflammation despite treatment. The role of HU in modulating inflammation in SCA remains uncertain. A previous study demonstrated that, while HU reduces the expression of the *NLRP3* gene, it does not affect the expression of other key inflammasome‐related genes such as *CASP1*, *IL1B* and *IL18*.^12^ Moreover, patients with SCA during vaso‐occlusive crises still exhibited increased neutrophil extracellular traps formation and a highly proinflammatory profile even during HU treatment.^13^ These data align with our observation that cytokines remain elevated in LU patients on HU therapy, underscoring the complexity of the inflammatory pathways involved in SCA.

The correlation analyses revealed several positive correlations among the cytokines across the groups, suggesting a coordinated pro‐inflammatory network. In patients with SCA and ALUs, a negative correlation between IL‐18 and Hb concentration, along with the inverse relationship between haemoglobin and LDH, underscores the link between systemic inflammation, haemolysis and impaired oxygen‐carrying capacity in patients with active ulcers. Additionally, the positive association between reticulocyte count and bilirubin further supports the presence of enhanced haemolytic activity in this group. These findings highlight the complex interplay between inflammation and haemolysis in the pathophysiology of leg ulcers in SCA.^14^, ^15^

Although inflammatory cytokines have been previously studied in SCA‐related LUs, our work provides additional insights into the systemic inflammatory environment linked to SCA ulcers through multivariate and correlation analyses. A key strength is the stratification by LU status, allowing a more detailed understanding of inflammatory dynamics across ulcer stages.

We acknowledge some limitations in our study. First, laboratory parameters and cytokine measurements were obtained at different time points, as laboratory data from the exact moment of blood collection were not available. About half of the LU group was on HU therapy. While excluding them would have substantially reduced the number of eligible patients, their inclusion allowed us to explore the influence of HU on the inflammatory profile of LU patients. Finally, a deeper characterisation of the ulcer microenvironment through sampling of cells at the ulcer site was not feasible, as most patients did not consent to this painful procedure.

In summary, our findings reinforce that LUs in SCA result from a multifactorial interplay between chronic inflammation, haemolysis and impaired wound healing. Elevated levels of cytokines, especially TNF‐α, in patients with both active and healed ulcers highlight the persistent inflammatory milieu associated with this complication, even post‐clinical resolution. Moreover, the persistent inflammation associated with LUs, even with HU therapy, suggests a limited impact of HU on cytokine‐mediated inflammation. Future research should focus on chronic inflammation mechanisms and inflammasome pathways to develop personalised, effective treatments for SCA patients with LUs.

## AUTHOR CONTRIBUTIONS

M.V.D. performed the experiments, performed the statistical analyses, interpreted the data and drafted the manuscript. A.P.S., B.R.S.B., C.M.L.M., F.O.S., J.V.S.R., G.S.A., H.I.L., M.J.B.M.R., R.C.S. and T.H.C.B. performed the experiments, updated the clinical data and interpreted the data. A.P.S., A.R.L.A., G.S.A. and I.F.D. updated the clinical data and reviewed the manuscript. A.C.M.A., A.P.S., A.S.A., G.S.A., M.A.C.B., M.V.D. and T.H.C.B. recruited the patients, assured access to patients' samples and updated the clinical data. S.T.O.S., F.F.C., A.R.L.A., G.S.A., I.F.D. and M.A.C.B. conceived and designed the study, analysed and interpreted data, and reviewed the manuscript. M.A.C.B. gave the final approval of the version to be submitted.

## FUNDING INFORMATION

This work was supported by Conselho Nacional de Desenvolvimento Científico e Tecnológico (CNPq, Grant #405918/2022‐4) and (CNPq, Grant #408710/2021‐7).

## CONFLICT OF INTEREST STATEMENT

The authors declare no competing financial interests.

## ETHICS STATEMENT

The local research ethics board approved this study (approval number 49177021.8.0000.5208), and following the Declaration of Helsinki, informed consent was obtained from all participants before the study commencement.

## Supporting information


Data S1.
