# Supplementary material for: Inflammatory cytokine profile in patients with sickle cell anaemia and leg ulcers
Source: Br J Haematol. 2025 Nov 3;208(1):373–7. doi: 10.1111/bjh.70236 (PMC12819091; doi:10.1111/bjh.70236)
Supplement: Supplementary file 1 — Data S1. [file BJH-208-373-s001.pdf]

SUPPLEMENTAL DATA

Inflammatory Cytokine Profile in Patients with Sickle Cell Anaemia and Leg Ulcers

Diniz, M.V.[1], Silva, A.P.[1], Arcanjo, G.S.[1], Batista, T.H.C.[1], Rodrigues, J.V.S.[1], Rêgo, M.J.B.M.[2], Leão, H.I.[2], Silva, R.C.[2], Souto, F.O.[3], Melo, C.M.L.[4], Barros, B.R.S.[4], Anjos, A.C.M.[5], Araújo, A.S. [5], Domingos, I.F.[6], Saad S.T.O.[7], Costa, F.F. [7], Araújo, A.R.L.[1], Bezerra, M.A.C.[1]*

[1]Genetics Postgraduate Program, Federal University of Pernambuco, Recife, Pernambuco, Brazil

[2]Center for Research in Therapeutic Innovation – NUPIT – UFPE, Recife, Brazil

[3]Aggeu Magalhães Institute/Oswaldo Cruz Foundation, Recife, Pernambuco, Brazil

[4]Laboratory of Immunological and Antitumor Analysis – LAIA, Keiso Asami Institute of Immunopathology – UFPE, Recife, Brazil

[5]Department of Internal Medicine, Hematology and Hemotherapy Foundation of Pernambuco, HEMOPE, Recife, Brazil

[6]Cardiology Emergency Unity of Pernambuco – PROCAPE - UPE, Recife, Brazil

[7]Hematology and Hemotherapy Center, State University of Campinas, Campinas, São Paulo, Brazil

***Corresponding Author:** Marcos A C Bezerra. Department of Genetics, Federal University of Pernambuco, Recife, Brazil. Av. Prof. Moraes Rego, 1235, Recife, PE 50670-901, Brazil. Tel: +55(81)2126-7825. Fax: +55(81)2126-7825

Email: macbezerra.ufpe@gmail.com

METHODS

Patients

Between March 2022 and February 2024, peripheral blood samples were collected from 76 individuals with SCA, all over 18 years of age, who were regularly monitored by a reference blood centre in Northeast Brazil. All patients were randomly recruited during routine medical visits. Patients receiving chronic blood transfusions, as well as those hospitalised for acute vaso-occlusive crises or related complications within the three months before blood collection, were excluded from the study. Based on the status of LUs, patients were classified into active LU (ALU), which included 23 SCA patients with ALU at the time of sample collection, and the healed LU (HLU), which included 22 SCA patients with previously diagnosed LUs that were fully healed by the time of sampling. At the time of blood sample collection, 55.6% (25/45) of patients with LUs were undergoing hydroxyurea (HU) therapy. Patients receiving HU therapy had been in treatment for at least one year at the maximum tolerated dose, ranging from 30 to 35 mg/kg/day.

The Control-HbSS consisted of 31 SCA patients with no history of LU, either before or during the study period. These individuals were not undergoing HU therapy due to unsatisfactory clinical response or personal reasons, such as procreation-related concerns, side effects, or drug intolerance. The Control-HbAA group consisted of 20 Afro-descendant blood donors recruited from the reference blood centre, with a normal haemoglobin profile (HbAA) confirmed by haemoglobin electrophoresis. These individuals had no history of diabetes or dermatological conditions such as ulcers, skin wounds, psoriasis, or atopic eczema at the time of sample collection. They also had no autoimmune diseases and were not experiencing any infectious processes during sample collection.

Clinical and baseline laboratory data (foetal haemoglobin levels, hematologic profile, and biochemical parameters) were obtained retrospectively from medical records. For patients under HU therapy, the laboratory data were obtained specifically from treatment-free periods. Homozygosity for haemoglobin S (HbSS) was confirmed in all SCA patients using high-performance liquid chromatography (HPLC) on the Variant II Turbo™ system (Bio-Rad Laboratories, Hercules, CA, USA). The Gap-PCR technique was used to determine the coinheritance with $^{-3.7Kb}$ alpha-thalassaemia deletion (1). The β^S-globin gene cluster haplotypes were also determined as previously described (2). The

study was approved by the local research ethics committee (approval number 49177021.8.0000.5208), and written informed consent was obtained from all participants prior to their inclusion in the study.

Detection of serum levels of IL-1β, IL-6, IL-8, IL-10, IL-12p70, IL-18 and TNFα

At the time of patients' recruitment, samples were collected in tubes without anticoagulant, subsequently centrifuged at 1,000g for 15 minutes, and the serum was stored at -80°C. The inflammatory cytokine IL-18 was measured using a commercial ELISA kit (QuantikineELISA Kits, R&D Systems, Minneapolis, MN, USA). The cytokines IL-1β, IL-6, IL-8, IL-10, IL-12p70 and TNFα were measured from serum samples using flow cytometry with a commercial human inflammatory cytokine kit (BD™ Cytometric Bead Array – CBA – Human InflammatoryCytokines Kit, Becton, Dickinson and Company. © 2008 BD).

Statistical analysis

Patient baseline characteristics were reported descriptively. Fisher's exact test or chi-square test, as appropriate, was used to compare categorical variables. Continuous variables were expressed as median and were compared using the Mann-Whitney or Kruskal-Wallis test, followed by Dunn's multiple comparisons post-test. To explore associations between cytokine levels and clinical or haematological parameters, Spearman correlation analysis was conducted within each group (Active LU, Healed LU, and Control HbSS). Correlation coefficients (r) and corresponding p-values were used to assess the strength and significance of relationships, with a significance threshold set at $p < 0.05$.

Multivariate analysis was performed using Partial Least Squares Discriminant Analysis (PLS-DA) to identify cytokine profiles that best distinguished among the clinical groups. Variable Importance in Projection (VIP) scores were calculated to rank cytokines based on their contribution to group separation. These analyses were conducted using MetaboAnalyst 6.0 (https://www.metaboanalyst.ca/). All statistical analyses were performed using SPSS Statistics 19.0 (IBM Corporation, Somers, NY, USA), GraphPad Prism version 6.0 (GraphPad Software, San Diego, CA, USA) and R software.

COMPLEMENTARY RESULTS

Patients' description

In the cohort, the median age was 36 years (range: 18–60), with 50% of participants being female. Out of the 76 SCA patients, 23 patients (30,3%) presented active LU, followed by 22 (28,9%) with healed LU and 31 patients (40,8%) with no clinical history of LU development. Haemoglobin F (HbF) levels ($p = 0.004$) and total haemoglobin (Hb) levels ($p = 0.014$) were significantly lower in the ALU group compared to others. A total of 52.7% of individuals had the β^S-globin CAR-CAR haplotype. No significant association was found between the α-thalassemia $^{-3.7kb}$ mutation (αα/-α or -α/-α) or β^S-globin haplotypes with LUs ($p > 0.05$). During the study recruitment, 55.6% (25/45) of patients with LUs were undergoing hydroxyurea (HU) therapy. Detailed clinical and laboratory data are presented in Table S1. The group control-HbAA was composed of 20 individuals with a median age of 36 years (range: 22-52, 50% males).

Cytokine levels in patients with leg ulcers versus controls

To investigate the inflammatory profile associated with LU in SCA patients, we first compared the serum levels of the cytokines among three groups: LU group (ALU and HLU), Control SS and Control AA (Figure S1 A-G). TNF-α levels (Figure S1B) were significantly elevated in the LU group compared to both Control SS ($p = 0.020$) and Control AA ($p = 0.0001$). IL-12p70 levels (Figure S1F) were significantly higher in the LU group compared to both Control SS ($p = 0.007$) and Control AA ($p = 0.0001$). IL-6 levels (Figure S1C) were also significantly increased in the LU group when compared to the Control AA ($p < 0.0001$), and the Control SS group exhibited higher IL-6 levels than those in the Control AA group ($p = 0.003$). Regarding IL-8 (Figure 1D), patients in the LU group displayed significantly higher levels compared to Control AA ($p = 0.0002$), although the differences between LU and Control SS were observed. IL-10 levels (Figure S1E) were significantly higher in LU patients compared to Control AA ($p = 0.035$). IL-18 levels (Figure S1G) were significantly elevated in the LU group compared to Control AA ($p < 0.0001$), while the differences between LU and Control SS and between Control SS and Control AA did not reach statistical significance. Finally, no significant differences in IL-

1β levels (Figure S1A) were observed among the three groups.

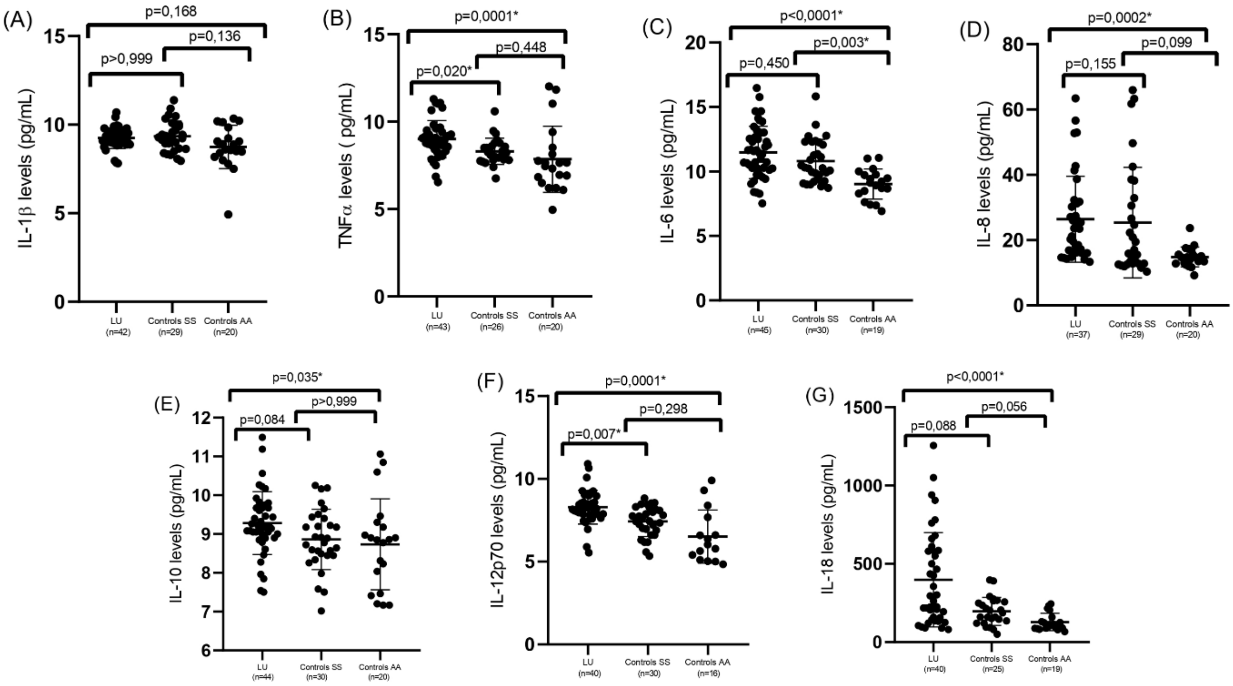

Fig. S1: Cytokine levels in patients with leg ulcers (LU) compared to control groups. Serum concentrations of IL-1β (A), TNF-α (B), IL-6 (C), IL-8 (D), IL-10 (E), IL-12p70 (F) and IL-18 (G) were measured and compared among patients with leg ulcers (LU), sickle cell anaemia controls without leg ulcers (Controls HbSS), and healthy controls with normal haemoglobin genotype (Controls HbAA). Data are presented as individual values with mean and standard deviation. Statistical comparisons were performed using the Kruskal-Wallis test followed by Dunn's multiple comparisons post-test. Exact p-values are indicated; $*p < 0.05$ was considered statistically significant.

Cytokine levels in LU patients (HU+ and HU-) versus controls SS

To determine the influence of HU therapy on cytokine profiles, LU patients were divided into those receiving HU (HU+) and those not receiving HU (HU−) and compared to HU-naive Control SS (Figure S2 A-G). Cytokine profiling revealed that LU patients, regardless of HU use, maintained elevated TNF-α and IL-12p70 levels compared to HU-naive HbSS controls, indicating persistent inflammation independent of HU therapy. Moreover, HU-treated patients exhibited higher IL-12p70 levels compared to HU-naive HbSS controls. HU-treated patients showed significantly higher levels of IL-10 compared to both untreated patients and controls. No significant HU-related differences were observed for IL-1β, IL-6, IL-8 and IL-18.

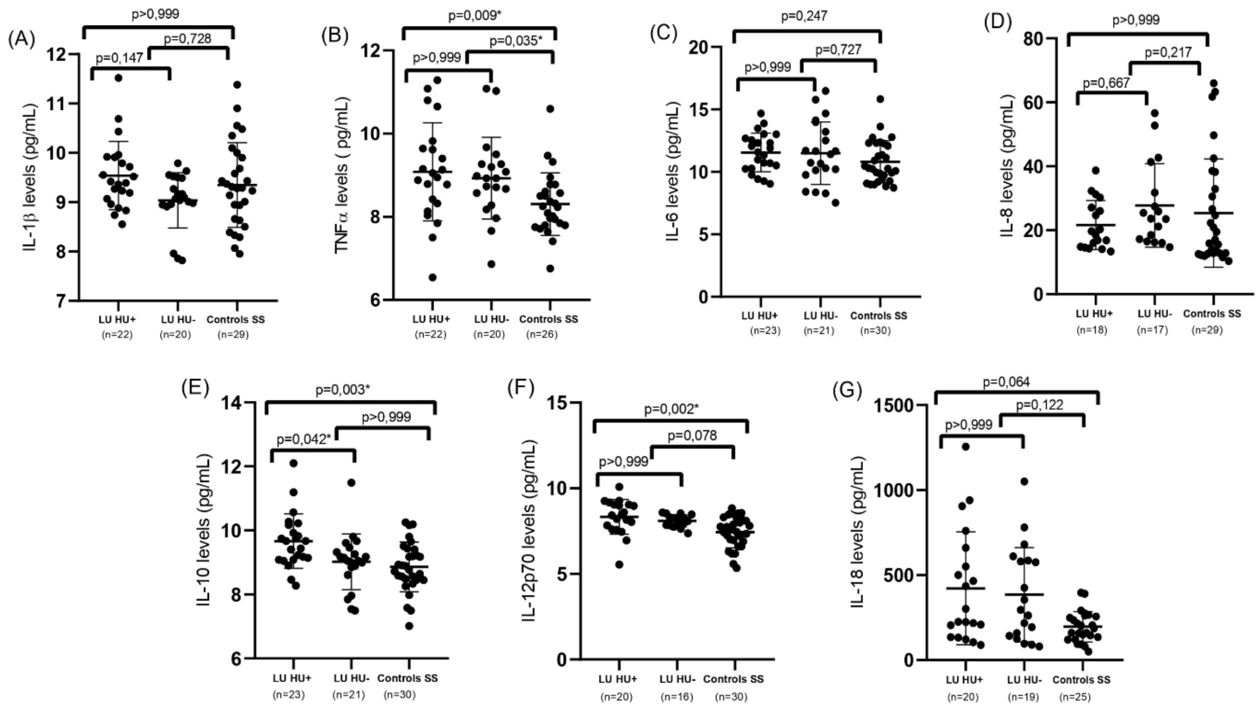

Fig. S2: Effect of hydroxyurea (HU) therapy on cytokine profiles in patients with leg ulcers (LU). Patients with LU were stratified based on hydroxyurea treatment status into HU-treated (LU HU+) and non-treated (LU HU-) groups and compared to hydroxyurea-naive sickle cell anaemia controls (Controls SS). Serum levels of IL-1β (A), TNF-α (B), IL-6 (C), IL-8 (D), IL-10 (E), IL-12p70 (F) and IL-18 (G) were assessed. Data are presented as individual values with mean and standard deviation. Statistical comparisons were performed using the Kruskal-Wallis test followed by Dunn’s multiple comparisons post-test. Exact p-values are indicated; *$p < 0.05$ was considered statistically significant.

SUPPLEMENTAL TABLE

Table S1. Baseline clinical and laboratory features of SCA patients according to the LU status.

Characteristics of patients	All patients (n = 76)		Active LU (n = 23)		Healed LU (n = 22)		Controls HbSS (n=31)		*P-value**
	n	%	n	%	n	%	n	%	
Sex									
Female	38	50	11	47,8	12	54,5	15	48,4	0,879
Male	38	50	12	52,2	10	45,5	16	51,6	
Age (years) Median	36		40		37		31		0,023*
Range	18-60		24-60		19-57		18-49		
VOCs/*per year*									
Missing data	4	5,3	0	0	1	4,5	3	9,7	
<3	34	44,6	9	39,1	9	40,9	16	51,6	0,508
3 - 6	25	8,0	8	34,8	8	36,4	9	29,0	
>6	13	6,0	6	26,1	4	18,2	3	9,7	
Hb (g/dL) median	7,8		7,0		7,6		8,5		0,014*
Hb F (%) median	5,8		4,0		8,0		7,2		0,004*
Reticulocyte (%) median	9,2		9,3		8,4		9,3		0,944
WBC (x 10^9/L) median	11590		12200		10500		11500		0,615
Platelets (x 10^9/L) median	402000		371500		402000		452000		0,565
TB (mg/dL) median	3,11		3,21		3,56		2,95		0,302
IB (mg/dL) median	2,30		2,08		2,85		2,28		0,482
LDH (U/L) median	753		938,5		822,5		544		0,119
Hydroxyurea therapy									
Missing data	3	3,9	0	0	0	0	3	9,7	
Yes	25	32,9	12	52,2	13	59,1	0	0	<0,0001*
No	48	63,2	11	47,8	9	40,9	28	90,3	
β^S haplotype									
Missing data	9	11,8	2	8,7	2	9,1	5	6,1	
CAR/CAR	40	52,7	13	56,5	9	40,9	18	8,1	0,244
Non-CAR/CAR	27	35,3	8	34,8	11	50,0	8	5,8	
α-thalassemia ($\alpha^{-3.7kb}$)									
Missing data	7	9,2	2	8,7	2	9,1	3	9,7	
Mutated	15	19,7	11	21,7	9	22,7	5	16,1	0,808
Non-mutated	54	71,1	12	69,6	13	68,2	23	74,2	

VOC, vaso-occlusive crisis; *RBC,* red blood cell; *Hb,* haemoglobin; *Hb F,* foetal haemoglobin; *WBC,* white blood cells; *TB*: total bilirubin; *IB,* indirect bilirubin; *LDH,* lactate dehydrogenase; CAR, CentralAfrican Republic. Laboratory parameters are described as median. Mutated alpha thalassemia are defined by one or two deletional α genes.

* Statistically significant difference ($p < 0.05$).

REFERENCES

1. Dodé C, Krishnamoorthy R, Lamb J, Rochette J. Rapid analysis of -α 3.7 thalassaemia and ααα anti 3.7 triplication by enzymatic amplification analysis. Br J Haematol. 1992 Jan;82(1):105–11.

2. Powars DR. Beta s-gene-cluster haplotypes in sickle cell anemia. Clinical and hematologic features. Hematol Oncol Clin North Am. 1991 Jun;5(3):475–93.
